# *Plasmodium falciparum* Choline Kinase Inhibition Leads to a Major Decrease in Phosphatidylethanolamine Causing Parasite Death

**DOI:** 10.1038/srep33189

**Published:** 2016-09-12

**Authors:** Lucía Serrán-Aguilera, Helen Denton, Belén Rubio-Ruiz, Borja López-Gutiérrez, Antonio Entrena, Luis Izquierdo, Terry K. Smith, Ana Conejo-García, Ramon Hurtado-Guerrero

**Affiliations:** 1 Department of Pharmaceutical and Organic Chemistry, c/ Campus de Cartuja, Faculty of Pharmacy, University of Granada, Granada, Spain; 2 Biomedical Sciences Research Complex, University of St Andrews, North Haugh, St Andrews, Fife, United Kingdom; 3 ISGlobal, Barcelona Centre for International Health Research (CRESIB), Hospital Clínic – University of Barcelona Barcelona, Spain; 4 Biosanitary Institute of Granada (ibs.GRANADA), SAS-Universidad de Granada, Granada, Spain; 5 BIFI, University of Zaragoza, BIFI-IQFR (CSIC) Joint Unit, Mariano Esquillor s/n, Campus Rio Ebro, Zaragoza, Spain; 6 Fundación ARAID, Zaragoza, Spain

## Abstract

Malaria is a life-threatening disease caused by different species of the protozoan parasite *Plasmodium*, with *P. falciparum* being the deadliest. Increasing parasitic resistance to existing antimalarials makes the necessity of novel avenues to treat this disease an urgent priority. The enzymes responsible for the synthesis of phosphatidylcholine and phosphatidylethanolamine are attractive drug targets to treat malaria as their selective inhibition leads to an arrest of the parasite’s growth and cures malaria in a mouse model. We present here a detailed study that reveals a mode of action for two *P. falciparum* choline kinase inhibitors both *in vitro* and *in vivo*. The compounds present distinct binding modes to the choline/ethanolamine-binding site of *P. falciparum* choline kinase, reflecting different types of inhibition. Strikingly, these compounds primarily inhibit the ethanolamine kinase activity of the *P. falciparum* choline kinase, leading to a severe decrease in the phosphatidylethanolamine levels within *P. falciparum,* which explains the resulting growth phenotype and the parasites death. These studies provide an understanding of the mode of action, and act as a springboard for continued antimalarial development efforts selectively targeting *P. falciparum* choline kinase.

Malaria remains an important global health concern. According to the latest estimates, 214 million cases of malaria occurred globally in 2014 and approximately half million deaths, primarily children aged under 5 year, in Africa^1^. Malaria is caused by various *Plasmodium* parasites. Of these, *P. vivax*, *P. malariae, P. ovale* and *P. falciparum* cause malaria in humans with the latter being one of the most common and the most deadly. In recent years, *P. knowlesi* has also been reported to infect humans in certain forested areas of South-East Asia^2^. A major obstacle to the eradication of this disease is the emerging resistance of *Plasmodium* parasites to most marketed antimalarial drugs^3^,^4^,^5^. There is therefore an urgent need for new antimalarial agents acting through novel mechanisms of action.

Phosphatidylcholine (PC) and phosphatidylethanolamine (PE) are major and essential phospholipids in *P. falciparum* membranes. Their content is unusually high representing 40–50% and 35–40% of the total phospholipids in *P. falciparum*, respectively^6^,^7^. Thus, the pathways leading to their synthesis are attractive targets for malaria chemotherapy^8^,^9^,^10^,^11^. PC and PE are mainly synthesized in *P. falciparum* by the Kennedy pathways from choline (Cho) or ethanolamine (Etn)^12^. The synthesis is initiated by phosphorylation of Cho and Etn to render phosphocholine (PCho) and phosphoethanolamine (PEtn) by choline kinase (CK) and ethanolamine kinase (EK) respectively. However, in many organisms one or other or both of these kinases are able to phosphorylate both substrates to varying degrees^12^. PCho and PEtn are in turn converted to CDP-choline and CDP-ethanolamine by the choline-phosphate cytidyltransferase (CCT) and ethanolamine-phosphate cytidyltransferase (ECT), respectively. Finally, PC and PE are synthesized *via* a common choline/ethanolamine phosphotransferase (CEPT). In addition, *P. falciparum* possesses an alternative route for the synthesis of PC from Etn that involves the methylation of PEtn by a phosphoethanolamine N-methyltransferase (PMT) to form PCho which subsequently form PC, thus connecting both Kennedy pathways^6^,^13^.

We have previously focused on the Cho phospholipid metabolism in cancer cells and tumors as a therapeutic target by inhibiting the human CK activity. In humans, CK is encoded by two genes, which express three isoforms CKα1, CKα2 and CKβ. An overexpression of the α-isoform of CK has been identified as an oncogene that mediates human cell transformation. As a consequence, CKα has been proposed and validated as a molecular target for the development of novel cancer therapeutic agents^14^,^15^. We rationally designed several CKα inhibitors initially based on structural modifications of the Cho uptake inhibitor Hemicholinium-3 and in the crystal structure of human CKα2 isoform subsequently^16^,^17^,^18^,^19^,^20^,^21^,^22^. Our earlier results showed one compound (BR7, Figure S1) that was uniquely able to occupy both Cho and ATP binding sites simultaneously^20^ whereas the rest of the inhibitors exclusively bound to the Cho-binding site^21^,^22^. We have also shown recently that compound BR33 (Figure S1) was able to induce local conformational changes in the Cho-binding site, which induced the aperture of an adjacent binding site^22^.

Considering their inhibition properties in the human CKα1 (*Hs*CKα1) and the high identity between the human CKα1 and the *P. falciparum* CK (*Pf*CK) at the binding site (29% identity between *Hs*CKα1 and *Pf*CK that increases up to ~69% when only conserved residues of ATP and Cho binding sites are considered (ref. 23; Figure S2), we rationalized that these compounds might also inhibit *Pf*CK, and therefore the growth of *P. falciparum* parasites. Thus, we recently evaluated the *in vitro* antiplasmodial and cytotoxic effects of some of these inhibitors against *P. falciparum*^24^. The compounds were found to be lethal for the blood stages of the parasite in a dose-dependent manner with IC_50_ values in the low nM range. The two most active compounds, BR23 and BR25 (Figure S1), with IC_50_ of 3 and 2 nM respectively^24^, were selected to further investigate their mode of action against *P. falciparum*.

Herein, we present a study on the mechanism of action of BR23 and BR25 both *in vitro* and *in vivo*. BR23 and BR25 lead to a severe drastic decrease of the PE pools *in vivo* that might explain the potent antiplasmodial and highly selective effect shown previously^24^. Contrary to what could be expected from the *in vivo* results, the compounds do not inhibit the *Pf*EK but the *Pf*CK. Surprisingly, our data reveals that these compounds have slightly different types of inhibition and majorly inhibit the EK activity of *Pf*CK. Finally, both compounds present two different binding modes in the Cho-binding site of *Pf*CK compared to the human enzyme.

## Results

### BR23 and BR25 lead to a dramatic decrease of the parasite PE pool

**Figure 1:**
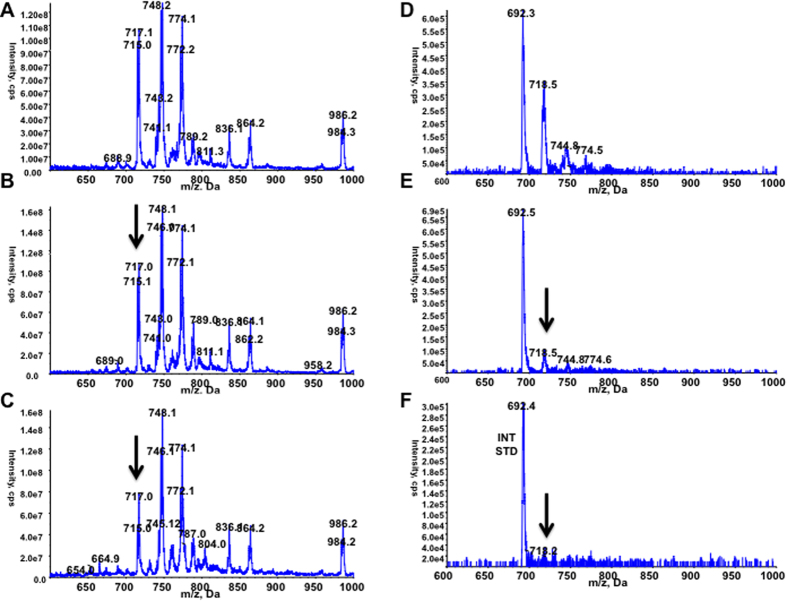
ES-MS and ES-MS-MS lipidomic analysis. Lipid extracts were made from *P. falciparum* in the absence (**A**) or presence of BR23 (**B**) and BR25 (**C**). Spectra show negative ion survey scans (600–1000 m/z). **PE analysis by ES-MS-MS.** Lipid extracts were made (spiked with the internal standard 1,2-dipalmitoyl-sn-glycero-3-phosphoethanolamine, 692 m/z) from *P. falciparum* in the absence (**D**) or presence of BR23 (**E**) and BR25 (**F**). Spectra show ES-MS-MS parents of 196 m/z (for PE lipid species) spectra over the mass range (600–1000 m/z).

As it was highly likely that treatment of either BR23 or BR25 to the whole parasite could affect phospholipid biosynthesis, namely the synthesis of PC and PE, we performed lipidomic analysis of parasites treated with the compounds and compared these to untreated. The positive-ion survey scan of the untreated cells revealed the expected array of PC species, which were unchanged in the lipid extracts of the cells treated with either BR23 or BR25 (Figure S3). The corresponding negative ion survey spectrum of the untreated cells showed the expected mixture of PE, phosphatidylserine PS, phosphatidylinositol (PI) and phosphatidylglycerol (PG) species (Fig. 1A) as previously described^25^. However, upon treatment with BR23, the relative amount of the lipid species at 715 and 717 m/z were decreased compared to the other phospholipids (black arrows within Fig. 1B). ES-MS-MS daughter ion fragmentation of these lipid species (Figure S4) revealed that they correspond to PE (34:1 and 34:2). The reduction in the PE species was more pronounced in the cells treated with BR25 at EC90 (black arrows within Fig. 1C), compared to the wild type after 3h treatment.

In order to confirm these observations and to quantify the reduction in PE species levels, new samples were treated at EC90 for 3 h with the inclusion of internal standard of PE and PC. Upon ES-MS-MS analysis looking at parents 196 m/z, which shows only the PE species (Fig. 1D–F), untreated cells showed the internal PE standard species (692 m/z), and other endogenous PE species at 715–719 m/z (34:0–2), 743–5 m/z (36:1–2) and 775 m/z (38:1) (Figure S4 and data not shown). Upon treatment with BR23, all of these endogenous PE species are greatly reduced (>90%), while the BR25 treated cells show an almost complete loss of all of the endogenous PE species (compare black arrows within Fig. 1E,F with control 1D). In contrast, treatments of BR23 or BR25 do not affect the level of PC species as determined by the parents of 184 m/z spectra (data not shown). We conclude that our compounds lead to a major decrease of the PE species without drastically affecting the PC species in the parasite.

### *P. falciparum* trophozoites are highly susceptible to the effect of both inhibitors

**Figure 2:**
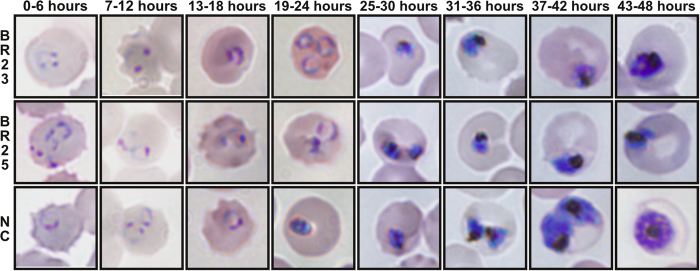
Microscopic effects (x1000 magnification) of BR23 and BR25 inhibitors on the asexual blood stages of a tightly (6 hours window) synchronized *in vitro* culture of *Plasmodium falciparum*. Negative control (NC) depicts representative stages of a complete intraerythrocytic cycle of *P. falciparum* in absence of inhibitors. BR23 and BR25 display the effects of the corresponding inhibitor at its EC_90_ concentration on morphology and development of the asexual blood stages. Time indicates hours post-invasion.

Prompted by the above results, we analyzed the global phenotypic effect of the massive reduction of the PE species in the parasite. Treatment of tightly synchronized ring stage parasites (6–12 hours post invasion) with EC_90_ concentrations of BR23 and BR25, lead to an arrest of parasite development. Rings seemed refractory to BR23 and BR25 inhibitory effects, whereas young trophozoites were highly susceptible (Fig. 2), in agreement with the increase of PE biosynthesis by the parasite in the second half of its asexual life cycle^26^. Arrested trophozoites did not progress into mature segmenting schizonts. Thus, a plausible explanation of the above experiments will involve the stalled trophozoites may be requiring PE for membrane biogenesis as well as for glycosylphosphatidylinositol (GPI) biosynthesis for numerous GPI-anchored proteins known to be essential at a schizont stage^11^,^27^.

### *Pf*CK determines the synthesis of all PCho and probably the majority of PEtn

To understand the molecular basis of the massive reduction in PE species, we rationalized that either the inhibition of *Pf*CK or *Pf*EK might explain the resulting phenotype induced by these compounds. While we selected *Pf*CK due to the previous demonstration that these compounds inhibited the human enzyme^22^, *Pf*EK was also taken into account because of the PE reduction-associated phenotype found in *P. falciparum*. At the ATP and Cho-binding sites, the similarities in sequence between both enzymes were high (26% identity between *Pf*CK and *Pf*EK that increases up to ~62% when only conserved residues of ATP and Cho-binding sites are considered; Figure S2), which are in agreement with their very similar catalyzed reactions. Hence, we inferred that either *Pf*CK or *Pf*EK enzymes might be drug targets for these compounds.

**Figure 3:**
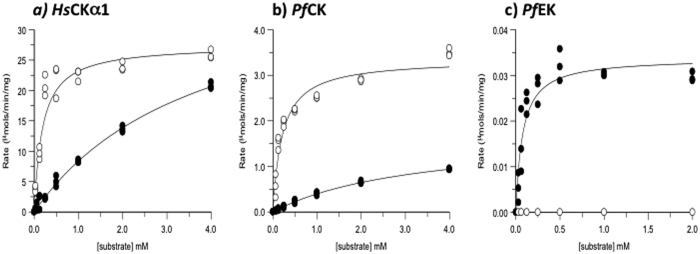
Cho and Etn saturation curves of recombinant *Hs*CKα1 (**A**), *Pf*CK (**B**) and *Pf*EK (**C**). ATP concentrations were held constant while Cho and Etn concentrations were varied as described in the Methods. Any ATPase activity was subtracted from the rates (*Pf*EK only) and all points were assayed in triplicate. The values shown as open and closed circles are determined in the presence of Cho and Etn, respectively.

Then, we conducted the determination of the kinetic parameters of the *Hs*CKα1, *Pf*CK and *Pf*EK enzymes (see Methods). Note that *Hs*CKα1 was also kinetically evaluated to compare its kinetic parameters with the *P. falciparum* enzymes. As expected and in agreement with the literature^18^,^28^,^29^, the *Hs*CKα1 showed a high specific activity and affinity for both Cho and ATP, which were reflected by a high kcat and catalytic efficiency (Fig. 3A and Table 1). As with many of the kinases that initiate the Kennedy pathway, the *Hs*CKα1 is able to phosphorylate both Cho and Etn^12^. However, while the K_m_ for Etn is ~20 times higher than the K_m_ for Cho, their kcats are comparable (Fig. 3A and Table 1).

Corresponding kinetic analysis of the *Pf*CK (Fig. 3B and Table 1) revealed that it was also able to phosphorylate both Cho and Etn. *Pf*CK behaved in a very similar manner to the human enzyme in terms of K_m_s whereas the specific activity was lower for the parasite enzyme (Table 1). The latter is reflected in the catalytic efficiency, which is 10–20 times lower than that of the human enzyme.

On the contrary, the *Pf*EK was specifically able to phosphorylate Etn and not Cho, and this was at a very low catalytic efficiency (Fig. 3C and Table 1). In fact, the *Pf*CK is able to phosphorylate Etn ~60 times faster than the *Pf*EK. Thus, depending upon the enzymes expression levels (and possibly cellular location), *Pf*CK is likely to be responsible for the phosphorylation of all the Cho and the vast majority of the Etn in the parasites Kennedy pathway.

### BR23 and BR25 inhibit EK activity of *Pf*CK with higher efficiency than CK activity

With the necessary kinetic values obtained, the inhibition of BR23 and BR25 on all three kinases was examined. Both of these compounds showed potent inhibition of the choline kinase activity of *Hs*CKα1 with IC_50_s of 940 and 520 nM, respectively (S1 Table). Rather surprisingly, given the high degree of sequence similarity (Figure S2) between *Hs*CKα1 and *Pf*CK, BR23 and BR25 only showed moderate inhibition towards *Pf*CKs choline kinase activity (S1 Table). However, when Etn was used as a substrate the inhibition of the kinase activity was far greater, with IC_50_s of 12 and 6 μM for BR23 and BR25, respectively (S1 Table). On the contrary, neither BR23 nor BR25 showed any inhibition towards *Pf*EK (S1 Table).

To further probe and understand the type of inhibition caused by BR23 and BR25 on *Hs*CKα1 and *Pf*CK, a series of kinetic analyses were undertaken to determine K_i_ values (S5–9 Figs). For *Hs*CKα1, BR23 and BR25 were shown to have mixed inhibition with respect to ATP, with K_i_s ~ 4 uM, and mixed inhibition with respect to Cho with potent K_i_s ~100 nM (Table 2). The calculated high alpha factors associated with the K_i_s against Cho, suggests that both BR23 and BR25 prevent binding of the substrate (Cho/Etn) and inhibition tends towards being competitive. For *Pf*CK, BR23 and BR25 also showed mixed inhibition against Cho with K_i_s ~35 uM, but in this case alpha factors were lower than for the human enzyme implying less impact on substrate binding. In contrast, both inhibitors were found to be competitive inhibitors of *Pf*CK with respect to Etn with K_i_s of ~4 uM (Table 2). This implies that the absolute binding positions of the two alternative substrates (Cho and Etn) must be different within the active site, leading to a different inhibition profile in presence of the inhibitors.

Collectively, this data clearly shows that both compounds behave differently depending upon the enzyme and substrate identity, thus causing a more potent inhibition of *Pf*CK when Etn is a substrate.

### BR23 and BR25 adopt different binding modes in *Pf*CK

To evaluate the affinity of BR23 and BR25 on *Hs*CKα1 and *Pf*CK, we determined the *K*_d_s by tryptophan fluorescence spectroscopy (Figure S10). Both compounds showed *K*_d_s in the low μM range with BR25 being 3-fold more potent in terms of affinity than BR23 for *Pf*CK (*K*_d_s for BR23 and BR25 were 1,400 ± 423 and 415 ± 81 nM, respectively). These values were also comparable to the obtained for the *Hs*CKα1 (*K*_d_s for BR23 and BR25 were 521 ± 200 and 352 ± 20 nM, respectively), in agreement with the high similarities inferred at the protein sequence level for both enzymes (Figure S2 and S2 Table).

**Figure 4:**
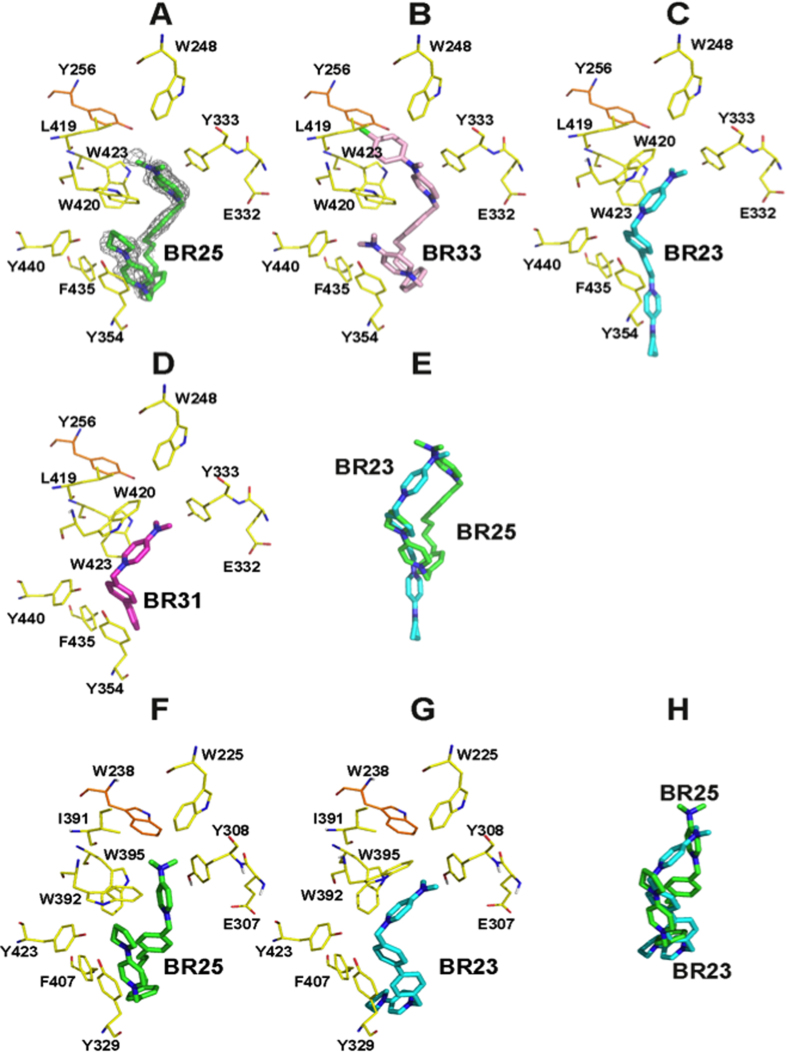
Active site of *Hs*CKα1 and *Pf*CK in complex with compounds BR23, BR25, BR33 and BR31. (**A**,**B**) Close-up view of compounds BR25 (PDB entry 5FUT) and BR33 (PDB entry 4CG8) bound to *Hs*CKα1, respectively. (**C**,**D**) Close-up view of compounds BR23 (obtained by docking studies, see Methods) and BR31 (PDB entry 4CG9) bound to *Hs*CKα1, respectively. (**E**) Superposition of compound BR23 and BR25. (**F**,**G**) Close-up view of compounds BR25 and BR23 bound to *Pf*CK (obtained by docking studies), respectively. (**H**) Superposition of bound compounds BR23 and BR25 within *Pf*CK Cho-binding site. Residues as shown as yellow carbon sticks except for the partial conserved residue that is in orange carbon sticks. The electron density map from the F_O_−F_C_ syntheses (grey) is contoured at 2.2 σ for BR25 (this density map is referred exclusively for BR25 in panel A). Note that we only report here the crystal structure of *Hs*CKα1 in complex with BR25. The others structures were published before (see PDB entries 4CG8 and 4CG9) or are obtained by docking studies (see Methods).

Prompted by the high binding affinities of BR23 and BR25, we decided to crystallize *Pf*CK in complex with both compounds to understand the molecular basis of how this enzyme recognizes these compounds. Despite of setting up a large number of crystallization trials, we were unable to obtain any crystals of *Pf*CK either apo- or in complex with either of the compounds. Knowing the high sequence similarity between *Pf*CK and *Hs*CKα1, we tried the alternative strategy to crystallize *Hs*CKα1 in complex with these compounds in order to understand their binding mode. Only crystals of *Hs*CKα1 in complex with compound BR25 were obtained that enabled us to solve the structure and unambiguously interpret the electron density maps (S3 Table). As expected, the crystal structure shows that BR25 is located within the known Cho-binding site (Fig. 4A). Strikingly the compound favors the unusual conformation also found for BR33 in which a new adjacent binding site is formed that is located at the back of the Cho-binding site (Fig. 4B)^22^.

Contrary to BR25, BR23 was computationally predicted to be located in the Cho-binding site of *Hs*CKα1 and adopt the typical binding mode, which is also visualized for most of the compounds previously crystallized such as BR31 (Fig. 4C,D; see Methods)^21^,^22^. Whereas compounds adopting the typical binding mode contain a short and rigid linker consisting of a biphenyl group, BR25 and BR33 have the flexible linker 1,4-biphenylbutane, explaining why the latter compounds adopt the unusual binding mode in which the aperture of an adjacent binding site is induced.

Compound BR25, in contrast to compound BR23, induces local conformational changes of several amino acids, allowing the 4-(dimethylamino)pyridinium fragment to access the adjacent binding site (Figs 4A and S11). This is mainly evidenced by W420 in which a dramatic conformational change occurs (Fig. 4A,C). W420 has to rotate <57.1° (the typical orientation of W420 found in most crystal structures is compared with the same amino acid from the complex with compound BR23) for the above fragment to access the hydrophobic pocket located in this adjacent binding site (Fig. 4A), which is formed by W248, Y256, Y333, L419, W420, and W423. The rest of BR25 is located in the Cho-binding site. The two positively charged fragments of BR25 forces the molecule to adopt a twisted conformation to maximize their interactions with the enzyme. While the 4-(dimethylamino)pyridinium fragment is characterized by a parallel π-cation interaction with Y333 and W420, respectively, the 4-pyrrolidinopyridinium fragment is tethered by Y354, W420, F435 and Y440 (Fig. 4A). Note that the 4-pyrrolidinopyridinium fragment establishes a parallel **π**-cation interaction with Y354 (Fig. 4A).

As expected, the benzyl-4-(dimethylamino)pyridinium fragment of compounds BR23 and BR31 superposes fairly well with an atomic shift of <0.30 Å, supporting the high similarities between both compounds (Figure S1). In particular, the biphenyl group of compounds BR23 and BR31 shows optimal parallel hydrophobic stacking interactions with Tyr354, and the 4-(dimethylamino)pyridinium moiety interacts through a parallel π-cation interaction with Trp420. The second positively charged fragment is exposed to the solvent in these types of compounds (Fig. 4C,D). A large RMSD of 7.6 Å between BR23 and BR25 also supports the large differences between these compounds in terms of their binding modes (Fig. 4E).

Both compounds were also computationally docked into *Pf*CK showing very similar features as found for the human enzyme (Fig. 4F–H). Again, BR23 adopted the typical binding mode whereas BR25 adopted the unusual conformation mentioned above. This is not surprising since the identity at the Cho-binding site between *Hs*CKα1 and *Pf*CK is high with only a minor difference between two hydrophobic residues located in the adjacent binding site (Y256 and W238 in the human and the parasite enzyme, respectively; Fig. 4F,G).

A large RMSD of 7.0 Å between BR23 and BR25 (Fig. 4H) might explain the different type of inhibition mainly when ATP varies (Table 2). Furthermore, both compounds also show different types of inhibition depending whether choline or ethanolamine is the substrate (Table 2), which also goes in line with both alternative substrates (Cho and Etn) adopting different binding modes in the Cho binding site.

## Discussion

**Figure 5:**
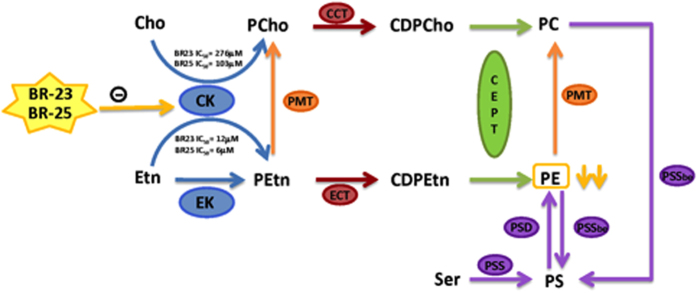
Mechanism of action for the inhibitors BR23 and BR25. Cho, choline; PCho, phosphocholine; CDPCho, cytidine diphosphocholine; PC, phosphatidylcholine, Etn, ethanolamine; PEtn, phosphoethanolamine; CDPEtn, cytidine diphosphoethanolamine; PE, phosphatidylethanolamine; Ser, serine, PS, phosphatidylserine; CK, choline kinase; EK, ethanolamine kinase, PMT phosphoethanolamine-N-methyltransferase, CCT, choline-phosphate cytidyltransferase; ECT, ethanolamine-phosphate cytidytransferase; CEPT, choline/ethanolamine phosphotransferase, PMT, phosphoethanolamine N-methyltransferase; PSS, phosphatidylserine synthase (CDP-DAG-dependent); PSSbe, phosphatidylserine synthase (via base exchange); PSD, phosphatidylserine descarboxylase.

This study set out to chemically validate either or both BR23 and BR25 as lead compounds that can effectively kill *P. falciparum* within red blood cells at low concentrations by preventing *de novo* synthesis of essential phospholipids PE and/or PC, i.e. by inhibiting the Cho/Etn kinases of the Kennedy pathway (Fig. 5). Various eukaryotic kinases that initiate the Kennedy pathway have been shown to be able to phosphorylate both Cho and Etn^12^. However, it has been suggested that *P. falciparum* has separate enzymes catalysing phophorylation of Cho and Etn^30^,^31^, although these authors state that redundancy remains a possibility. Here we show that the *Hs*CKα1 is able to phosphorylate both Cho and Etn, with comparable kcats, despite a significantly preference (lower K_m_) for Cho in accordance with previous studies^12^. Likewise *Pf*CK, despite its lower specific activity, is also able to phosphorylate both Cho and Etn with similar K_m_s to the human enzyme. The *Pf*EK enzyme has a very low catalytic efficiency (~60 times less that *Pf*CK) to phosphorylate Etn and showed no ability to phosphorylate Cho at all. This clearly shows that *Pf*CK is potentially responsible for the phosphorylation of all the Cho and likely the major source of PE in the parasites for the Kennedy pathway leading to PC and PE, respectively. The overall pool of PC and PE would be also dependant upon the enzymes’ expression levels and possibly cellular location. However, both are expressed at similar levels according to PlasmoDB, and are located in the cytosol, suggesting that these two latter factors would not be the rate-limiting step for the synthesis of PC and PE, the later being essential for GPI biosynthesis and maintaining the correct membrane composition, fluidity for numerous cellular processes and organelle function. Furthermore, it is obvious that the phosphoethanolamine N-methyltransferase acting upon PE is not the major source of PC as these levels remain relatively unchanged. Also the relative available concentration of Cho and Etn is crucial for the final effects of the inhibitors. In particular, intracellular Etn has been shown to be lower^32^, thus making Etn dependent enzymes more vulnerable to competitive inhibitors.

The potential inhibitory effect of BR23 and BR25 on both kinases was examined in detail by enzyme kinetics. Both BR23 and BR25 showed mixed inhibition of *Hs*CKα1 with respect to ATP and Cho, with K_i_s~4 uM, and K_i_s~100 nM respectively. The latter suggests that both compounds are competitive with respect to preventing the binding of the substrate (Cho/Etn). From the high sequence similarity between *Hs*CKα1 and *Pf*CK especially within the active site, it is not surprising that BR23 and BR25 also showed mixed inhibition against *Pf*CK for Cho (K_i_s ~35 uM), but compared to the human enzyme having less impact on Cho binding. However, both inhibitors were found to be competitive inhibitors of *Pf*CK with respect to Etn with K_i_s of ~4 uM. This implies that the absolute binding positions of the two alternative substrates (Cho and Etn) are different within the active site of *Pf*CK. This is supported by the crystal structure of *Hs*CKα1 with BR25, where the compound adopts an unusual binding mode that induces the aperture of an adjacent binding site located at the back of the Cho-binding site. However, BR23 and related analogues with a short and rigid linker consisting of a biphenyl group were computationally predicted and experimentally proven to adopt a typical binding mode within the Cho-binding site of *Hs*CKα1^21^,^22^.

When both compounds were computationally docked into *Pf*CK, their respective binding modes were very similar to those found for the human enzyme where BR23 adopts the typical binding orientation and BR25 adopted the unusual conformation mentioned above. Collectively, this data clearly shows that both compounds behave differently depending upon the enzyme and substrate identity, thus causing a more potent inhibition of *Pf*CK when Etn is a substrate. A better understanding and exploitation of this adjacent binding mode near the Cho binding site, may lead to greater potency and selectivity. While there is a Tyr (Y256) in the human enzyme, a Trp (W238) is present in the parasite enzyme (Fig. 4), which could be exploited to make selective compounds and could provide partially a plausible explanation for the selective nature of these compounds compared to mammalian cells and their respective C/E kinases^24^.

Despite the fact that neither BR23 nor BR25 showed any inhibition towards *Pf*EK, they did show significant inhibition of *Pf*CK ethanolamine kinase activity, thus suggesting that they might affect the availability of PEtn to be used by the downstream enzymes of the Kennedy pathway for the *de novo* biosynthesis of PE, which ends up being lethal to the parasite. The observed differences between enzyme and parasite inhibition can be explained in a variety of ways: (a) the compounds may accumulate within the parasite, hence reaching a higher local concentration; and (b) the accumulative effect of inhibiting PE biosynthesis even at only 10–20% over a period of 24 hours might cause a significant decrease in the pool of PE that is lethal to the parasite. The latter may be especially true during periods of rapid cell growth/division when the demand on PE is high as will be the flux of PE biosynthesis.

Indeed quantitative lipidomic analysis of parasites treated with BR23 showed a significant reduction in the parasites PE species, while treatment with BR25 showed an almost complete loss of all of the endogenous PE species. In stark contrast, treatments of BR23 or BR25 do not affect on the PC species significantly, highlighting again the differential inhibition of these compounds of *PfCK* in the presence of either Etn or Cho as substrate. This also highlights that both *Pf*EK, which only phosphorylates Etn, and the possible decarboxylation of PS (Fig. 5), are unable to compensate for the lack of EK activity of the *Pf*CK.

The reduced synthesis of PE in the parasites cause a number of detrimental issues that include the inability to synthesise mature GPI anchors and changes in the membrane composition (as it is a major lipid within the parasite). Inhibition of GPI synthesis disrupts the anchoring of essential cell-surface proteins to the membrane (i.e. MSP-1), as PE is the PEtn donor that links the proteins via their C-terminus to the third mannose of the GPI^33^. PE decrease also causes changes in membrane’s fluidity, which may also alter membrane potentials, leading to an impact on the functions within the mitochondria and potentially the apicoplast^11^.

In summary we have shown the following conclusions: (1) *Pf*CK is responsible for producing the vast majority of the PEtn used by the parasite to make PE; (2) the type of inhibition by BR23 and BR25 on *Pf*CK are influenced differentially by the presence of the alternative substrates, Etn or Cho; (3) the direct action of BR23 and BR25 on the parasites is to inhibit the formation of PEtn directly reducing the *de novo* biosynthesis of PE *via* the Kennedy pathway, which is ultimately lethal to the parasite; and (4) BR23 and BR25 are lead compounds with potent and selective nanomolar antiplasmodium activity that have been chemically validated against *Pf*CK.

## Materials and Methods

### Cloning and purification of *Hs*CKα1, *Pf*CK and *Pf*EK

Details about cloning and purification of human CKα1 and *Pf*CK have been previously reported^20^,^23^.

The DNA sequence encoding amino acid residues 2–420 of *Pf*EK, defined as *pfek*, was made synthetically and codon optimized by GenScript to be expressed in *E. coli*. *pfek* was amplified using the forward primer,

5′-CGGAATTC*CATATG*GAATACCAACTGCGTGAAATTG-3′ containing a NdeI site (italic), and the reverse primer

5′-CGGAATTC*GTCGAC*TCACAGTTTGCTGCGGAATTTCAC-3′ containing a SalI site (italic) and a stop codon (underlined). Subsequently the PCR product was digested with NdeI and SalI and cloned into a modified pET15b vector containing a histidine tag and a PreScission protease (PP) cleavage site, resulting in the expression plasmid pET15bPP-*pfek*. The plasmid was verified by sequencing.

pET15bPP-*pfek* was transformed into BL21 DE3 (star) cells grown in 2XYT medium with 100 μg/ml of ampicillin. Cells were grown at 37 °C until reaching an OD of 0.6 at 600 nm, after which the expression of the protein was induced overnight by 1 mM IPTG at 18 °C and 180 rpm.

The cells were harvested by centrifugation at 3480 g for 30 min and suspended in buffer A (20 mM phosphate, 500 mM NaCl, 10 mM imidazole, pH 7.4), containing lysozyme (1 mg/ml)), DNAse (0.1 mg/ml) and a cocktail of protease inhibitors (0.2 mM PMSF, 10 mM benzamidine and 0.5 mM leupeptin). The cells were disrupted by sonication and centrifuged at 19500 g at 4 °C for 30 minutes.

The supernatants were applied onto a 1 × 5 ml cobalt HP column (GE Healthcare) previously equilibrated with buffer A. The column was washed with buffer A and the fusion protein was eluted with buffer A containing 500 mM imidazole. The fusion protein was dialysed with buffer B (25 mM Tris/HCl, 150 mM NaCl pH 7.5) and subsequently was cleaved overnight with PP (GE Healthcare) at 4 °C. The PP was removed by a GSTrap column and the unbound untagged protein was applied onto a 1 × 5 ml cobalt HP column (GE Healthcare) previously equilibrated with buffer B. The unbound protein was further purified by size exclusion chromatography using a Superdex 75 XK26/60 column previously equilibrated with two volumes of buffer B. The eluted peaks were concentrated and used for biophysical experiments. The purity of the recombinant *Pf*EK was assessed by SDS-PAGE. *Pf*EK concentrations were determined spectrophotometrically using the extinction coefficient at 280 nm of 1.129 mg ml^−1^ cm^−1^. Finally the protein was stored in buffer B at −80 °C.

### *P. falciparum* culture and microscopy

*P. falciparum* 3D7 was cultured according to standard methods^34^, and a 6 hours window was achieved by a combination of Percoll and sorbitol synchronizations^35^. Briefly, schizonts and late trophozoites were isolated from earlier stages according to their density by centrifugation on a continuous gradient of 60% Percoll. The resulting schizonts were allowed to invade uninfected red blood cells for 6 hours and newly infected red blood cells were subjected to a 5% sorbitol treatment for 10 minutes at 37 °C. Parasitemia was adjusted to ≈0.5% and either BR23 or BR25 inhibitors were inoculated at their EC_90_s) after 6 additional hours of incubation (*i.e.* 6–12 hours post-invasion). Thin blood smears were Giemsa stained every 6 hours until an entire intraerythrocytic cycle was completed.

### *P. falciparum* lipid extraction

*P. falciparum* 3D7 were cultured with BR23 or BR25 inhibitors at their EC_90_s for 3h after sorbitol synchronization. After washing, *P. falciparum* trophozoites were cultured for 18h without inhibitors, collected, treated with saponin to release the parasites from the red blood cells, and washed twice with PBS prior to Bligh & Dyer lipid extraction^36^. Briefly, 5 × 10^8^ parasite cells were dissolved in 375 μl 1:2 (v/v) chloroform:methanol and vigorously vortexed for 15 min. 125 μl of chloroform were added and 500 pmols of 1,2-dimyristoyl-*sn*-glycero-3-phosphocholine and 1,2-dipalmitoyl-*sn*-glycero-3-phosphoethanolamine were included in each sample as internal controls. 125 μl of water were added to the sample, vortexed and centrifuged at ×1000 g (RT) for 5 min to make the sample biphasic. Lower organic phase was collected and upper (aqueous) phase was re-extracted twice with 125 μls of chloroform. Lipid extracts were pooled, dried under nitrogen and stored at 4 °C for ESI-MS/MS analysis.

### Assays for CK and EK enzyme activites

A continuous spectrophotometric 96-well plate assay was established for CK and EK activity based on an assay described^37^. The standard assay reaction contained, in a final volume of 100 μl: 50 mM MOPS, pH 7.8, 150 mM KCl, 6 mM MgCl_2_, 1 mM PEP, 1 mM NADH, 6 U/mL pyruvate kinase, 9 U/mL lactate dehydrogenase, 4 mM Cho or Etn, 2 mM ATP (for *Pf*EK) or 4 mM ATP (for CKs) and 0.1–50 μg enzyme. The reaction was initiated by addition of enzyme or substrate, as appropriate, and the rate of reaction was monitored by following the decrease in absorbance at 341 nm for 10 min at room temperature using a Thermo Multiska spectrophotometer. Experiments were carried out to ensure that coupling enzymes were not rate-limiting under any of the conditions used and control experiments lacking substrates and/or enzyme were carried out to correct for any background rate.

### Kinetic analyses

One substrate was held constant at the concentration specified above while the second substrate was varied. Any ATPase activity was controlled for and subtracted from the rates (*Pf*EK only). Curves were plotted and K_m_ and V_max_ values estimated using Grafit 5 (Erithacus Software Ltd).

### IC_50_ determinations

The enzyme was pre-incubated for 5 min with varying concentrations of inhibitor prior to reaction being started by addition of 0.5 mM each substrate. The IC_50_ values were estimated using Grafit 5 (Erithacus Software Ltd).

### K_i_ determinations

Activity was measured at 3–4 different concentrations of inhibitor and a fixed concentration of one substrate (as specified in the standard assay) while the second substrate was varied. GraphPad Prism 6 (GraphPad Software Inc.) was used to fit data to different inhibition models with the best fit being determined by Aike’s Informative Criteria. K_i_s were estimated from the best-fit model and results were reinforced by linear re-plots of the data (Lineweaver Burke, Dixon, s/v against I)^38^.

### Electrospray-mass spectrometry analysis

Lipid extracts, were dissolved in 15 μl of choloroform:methanol (1:2) and 15 μl of acetonitrile:iso-propanol:water (6:7:2) and analysed with a Absceix 4000 QTrap, a triple quadrupole mass spectrometer equipped with a nanoelectrospray source. Samples were delivered using a Nanomate interface in direct infusion mode (~125 nl/min). The lipid extracts were analysed in both positive and negative ion modes using a capillary voltage of 1.25 kV. MS/MS scanning (daughter, precursor and neutral loss scans) were performed using nitrogen as the collision gas with collision energies between 35–90 V. Each spectrum encompasses at least 50 repetitive scans. Tandem mass spectra (MS/MS) were obtained with collision energies between 35–90 V. Assignment of phospholipid and neutral lipid species is based upon a combination of survey, daughter, precursor and neutral loss scans, as well previous assignments^25^,^39^. The identity of phospholipid peaks was verified using the LIPID MAPS: Nature Lipidomics Gateway (www.lipidmaps.org).

### Docking studies

Whereas Maestro 9.3 software^40^ has been used for docking studies, Pymol^41^ has been used for preparing the figures that represent the obtained poses. PDB entries 4CG9^22^ and 3FI8 have been selected as templates for the docking studies of compounds BR23 and BR25 in *Hs*CKα1 and *Pf*CK. The PDB entry 4CG9 has been used for predicting the docking pose of compound BR23 in *Hs*CKα1 because it contains compound BR31^22^, which is an analog of compound BR23.

In a first step, all the proteins were aligned using the Protein Structure Alignment tool included into Maestro 9.3. Then, Protein Preparation Wizard module^42^ was used for preparing the protein structures. A pre-process was made adding hydrogen atoms and assigning bond orders. The PROPKA script^43^,^44^ was used to assign the correct tautomeric and protonation state of the amino acids at pH 7.0 ± 1.0 for optimizing H-bonds networks. Finally, an initial restrained minimization using the OPLS2500 force field and a convergence RMSD of 0.3 Å for heavy atoms were performed in order to optimize the protein and to assign the partial atomic charge to each atom. A potential map for PDB entries 4CG9 (*Hs*CKα1) and 3FI8 (*Pf*CK) were generated using the Shrödinger Glide module^45^. Compounds BR31 and BR25 were imported into *Pf*CK Cho-binding site as a reference for the Grid generation, being the Grid box centered in these molecules and defined with a high volume (25 Å) to allow the insertion of compounds BR23 and BR25. Once the 3D structures were prepared for each compound (whereas BR23 was obtained after adding the 4-pyrrolidinopyridine moiety to compound BR31, we used the BR25 structure visualized in the crystal structure), each molecule was optimized by means of the MacroModel module using the OPLS 2500 force field and a convergence gradient of 0.05 Å. After minimization, the LigPrep module^46^ was used for preparing the ligands for docking studies generating all possible conformers and protonation states at pH = 7.0 ± 1.0.

Finally, Glide was used to flexibly dock compounds BR23 and BR25 into the active site of both proteins (4CG9 and 3FI8 pdb codes) with extra precision, and BR25 into *Pf*CK binding pocket (3FI8) in the same conditions.

### Tryptophan fluorescence

Compounds BR23 and BR25 were prepared in 100% DMSO. The K_d_s for compounds BR23 and BR25 against *Hs*CKα1 and *Pf*CK were measured by monitoring the quenching of tryptophan fluorescence. All experiments were carried out in a Cary Eclipse spectrofluorometer at 25 °C with the enzymes at 1 μM, and concentrations of compounds varying from 0.05 to 8 μM for *Hs*CKα1, and from 0.05 to 16 μM for *Pf*CK in 25 mM Tris, 150 mM NaCl, pH 7.5. Fluorescence emission spectra were recorded in the 300–400 nm range with an excitation wavelength of 280 nm, with slit width of 5 nm. Controls were determined by incubating the enzymes with equivalent amounts of DMSO. As indicated previously, data analysis was performed in Prism (GraphPad software) considering a model with a single binding site (Eq. 1), where F0 is the intrinsic fluorescence of the enzyme in the absence of quencher (Q), F1 is the observed fluorescence at a given quencher concentration, fa is the fractional degree of fluorescence, and K_d_ is the dissociation constant.


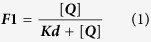


### Protein crystallization

*Hs*CKα1 at 20 mg/ml in buffer 25 mM Tris/HCl, 150 mM NaCl pH 7.5 was preincubated at room temperature with 12 mM of compound BR25 (DMSO is at 5% final concentration in the mix). The sitting-drop vapour diffusion method^47^ was used to produce crystals by mixing 0.5 μl of the protein solution and an equal volume of mother liquor (20% PEG 5000 MME and 0.2 M KSCN). Tetragonal crystals (space group P4_3_2_1_2) grew within 2–4 days and then were soaked for one day with 20 mM compound BR25 (the final concentration of DMSO during the soaking was ~5%). The crystals used in this study were cryoprotected in the previously stated mother liquor solution containing an additional 20% ethylenglycol and frozen in a nitrogen gas stream cooled to 100 K.

### Structure determination and refinement

Diffraction data of the binary complex was collected at XALOC (ALBA, Barcelona). The data was processed and scaled using the XDS package^48^ and CCP4 software^49^. The structure of the binary complex was solved by molecular replacement (MOLREP)^50^ using PDB entry 4CG9^22^ as a template. Initial phases were further improved by cycles of manual model building in Coot^51^ and restrained refinement with REFMAC5^52^. Finally, additional cycles of model building in Coot with TLS refinement in PHENIX^53^ were performed. The final model was validated with PROCHECK^54^) and relevant statistics are given in S3 Table. The topology for BR25 was generated with PRODRG^55^. Coordinates and structure factors have been deposited in the Worldwide Protein Data Bank (wwPDB; see S3 Table for the pdb code).

## Additional Information

**How to cite this article**: Serrán-Aguilera, L. *et al*. *Plasmodium falciparum* Choline Kinase Inhibition Leads to a Major Decrease in Phosphatidylethanolamine, Causing Parasite Death. *Sci. Rep.* **6**, 33189; doi: 10.1038/srep33189 (2016).

## Supplementary Material

Supplementary Information

## Figures and Tables

**Table 1:** Kinetic values for recombinant *Hs*CKα1, *Pf*CK and *Pf*EK.

Substrate	Cho				Etn				ATP
	V_max_ (μmols/min/mg)*	K_m_ (mM)*	kcat (s^−1^)	kcat/k_m_ (s^−1^mM)	V_max_ (μmols/min/mg)*	K_m_ (mM)*	kcat (s^−1^)	kcat/k_m_ (s^−1^mM)	K_m_ (mM)*
*Hs*CKα1	27.5 ± 1.4	0.19 ± 0.04	24	127	40.3 ± 2.6	3.8 ± 0.4	36	9.4	0.32 ± 0.05
*Pf*CK	3.3 ± 0.1	0.21 ± 0.03	2.9	13.8	1.7 ± 0.09	3.3 ± 0.3	1.5	0.45	0.13 ± 0.01
*Pf*EK	n.d.	n.d.	n.d.	n.d.	0.03 ± 0.002	0.08 ± 0.02	0.025	0.31	0.07 ± 0.03

^*^Mean ± standard deviation (n = 3), n.d., not detected.

**Table 2:** Modes of inhibition and K_i_ values for inhibition of recombinant *Hs*CKα1, *Pf*CK and *Pf*EK by BR23 and BR25.

Inhibitor	*Hs*CKα1			*Pf*CK		
	Cho	Etn	ATP	Cho	Etn	ATP
BR23	Mixed K_i_, 0.114 ± 0.020 μM α, 59	N.D.	Mixed K_i_, 3.7 ± 0.8 μM α, 0.73	Mixed K_i_, 38.2 ± 12.1 μM α, 3.915	Competitive K_i_, 4.1 ± 0.2 μM	Competitive K_i_, 116 ± 15 μM
BR25	Mixed K_i_, 0.100 ± 0.015 μM α, 139	N.D.	Mixed K_i_, 4.1 ± 1.2 μM α, 0.49	Mixed K_i_, 31.9 ± 5.5 μM α, 2.025	Competitive K_i_, 4.0 ± 0.1 μM	Mixed K_i_, 65.5 ± 5.7 μM α, 2.37

Means ± standard deviation (n = 3). N.D., not determined.
