# Supplementary material for: Plasmodium falciparum Choline Kinase Inhibition Leads to a Major Decrease in Phosphatidylethanolamine Causing Parasite Death
Source: Sci Rep. 2016 Sep 12;6:33189. doi: 10.1038/srep33189 (PMC5018819; doi:10.1038/srep33189)
Supplement: Supplementary Information [file srep33189-s1.docx]

**SUPPLEMENTAL DATA**

***Plasmodium falciparum* Choline Kinase Inhibition Leads to a Major Decrease in Phosphatidylethanolamine Causing Parasite Death**

Lucía Serrán-Aguilera^¶^, Helen Denton ^¶^, Belén Rubio-Ruiz, Borja López-Gutiérrez, Antonio Entrena, Luis Izquierdo, Terry K. Smith*, Ana Conejo-García*, and Ramon Hurtado-Guerrero*

**SUPPLEMENTAL FIGURES**

**S1 Fig. Chemical structures of the CK inhibitors mentioned in this work.**





**S2 Fig. Multiple sequence alignments of *Hs*CKα1, *Pf*CK and *Pf*EK.** Residues located in the ADP and Cho-binding site are highlighted in yellow and blue, respectively. Green triangles denote the conserved residues, whereas red circles represent the residues that interact with substrate through hydrophobic interactions or saline bonds.


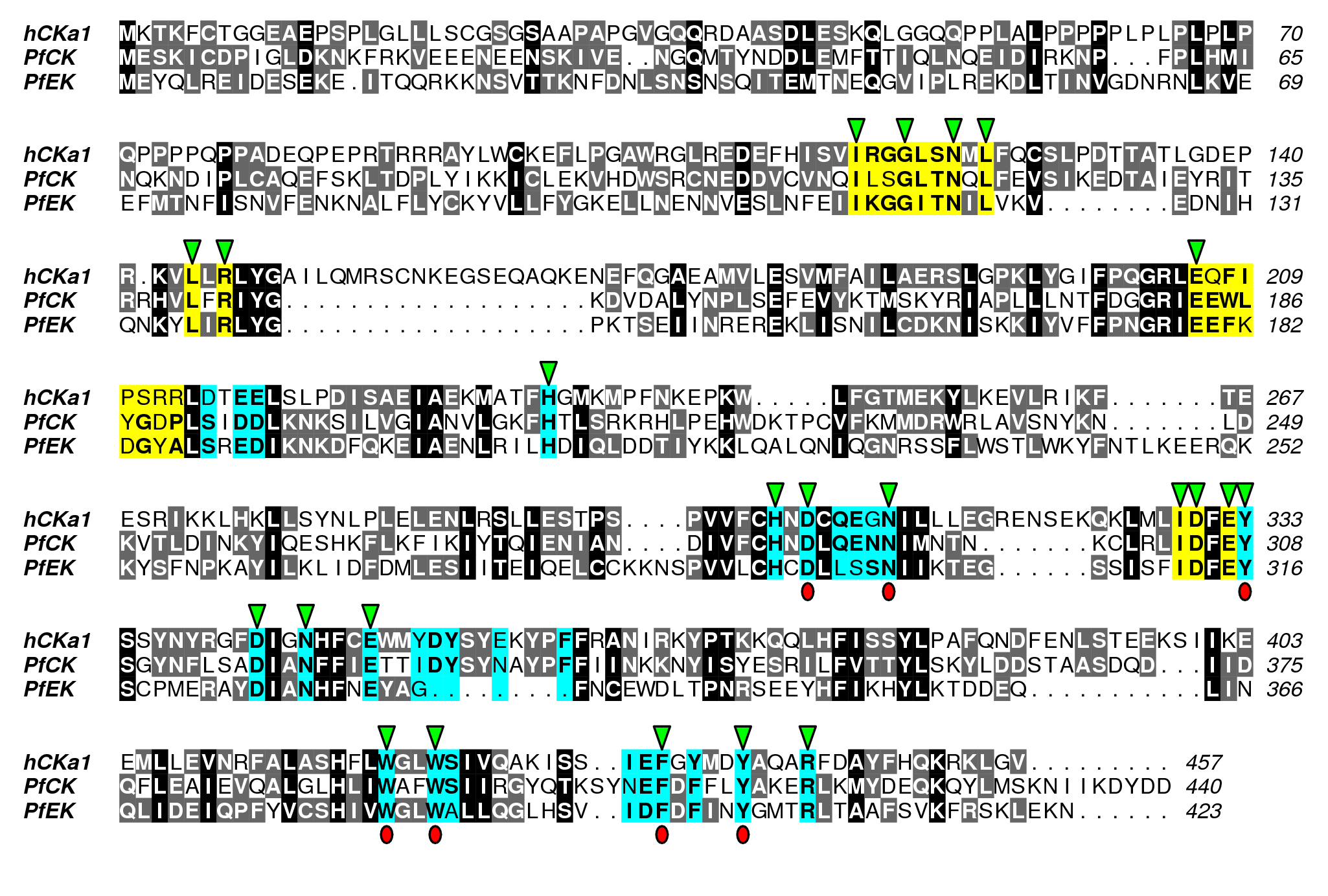


**S3 Fig. ES-MS positive ion survey lipidomic analysis. Lipid extracts were made from *P. falciparum* in the absence (A) or presence of BR23 (B) and BR25 (C).** Spectra show positive ion survey scans (600-1000m/z).


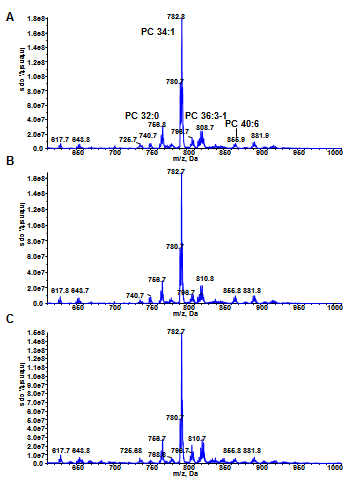


**S4 Fig. ES-MS-MS negative ion daughter ion spectra of PE species.** Lipid extracts from *P. falciparum* as described in experimental section. Daughter ion spectra of A) 717 m/z B) 715 m/z and C) 743 m/z, clearly show the major fragments including the acyls group attached to the PE species, i.e. 281 m/z is C18:1, 279 m/z is 18:2, 255 is C16:0.


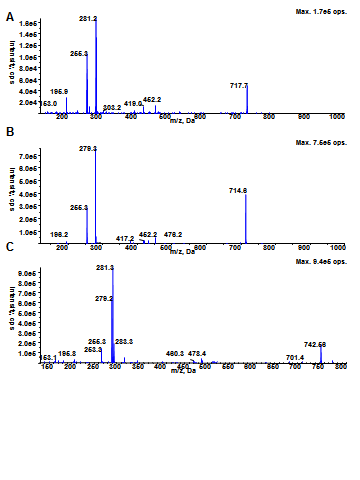


**S5 Fig. Inhibition kinetics of *Hs*CKα1 by BR23, Cho as variable substrate**


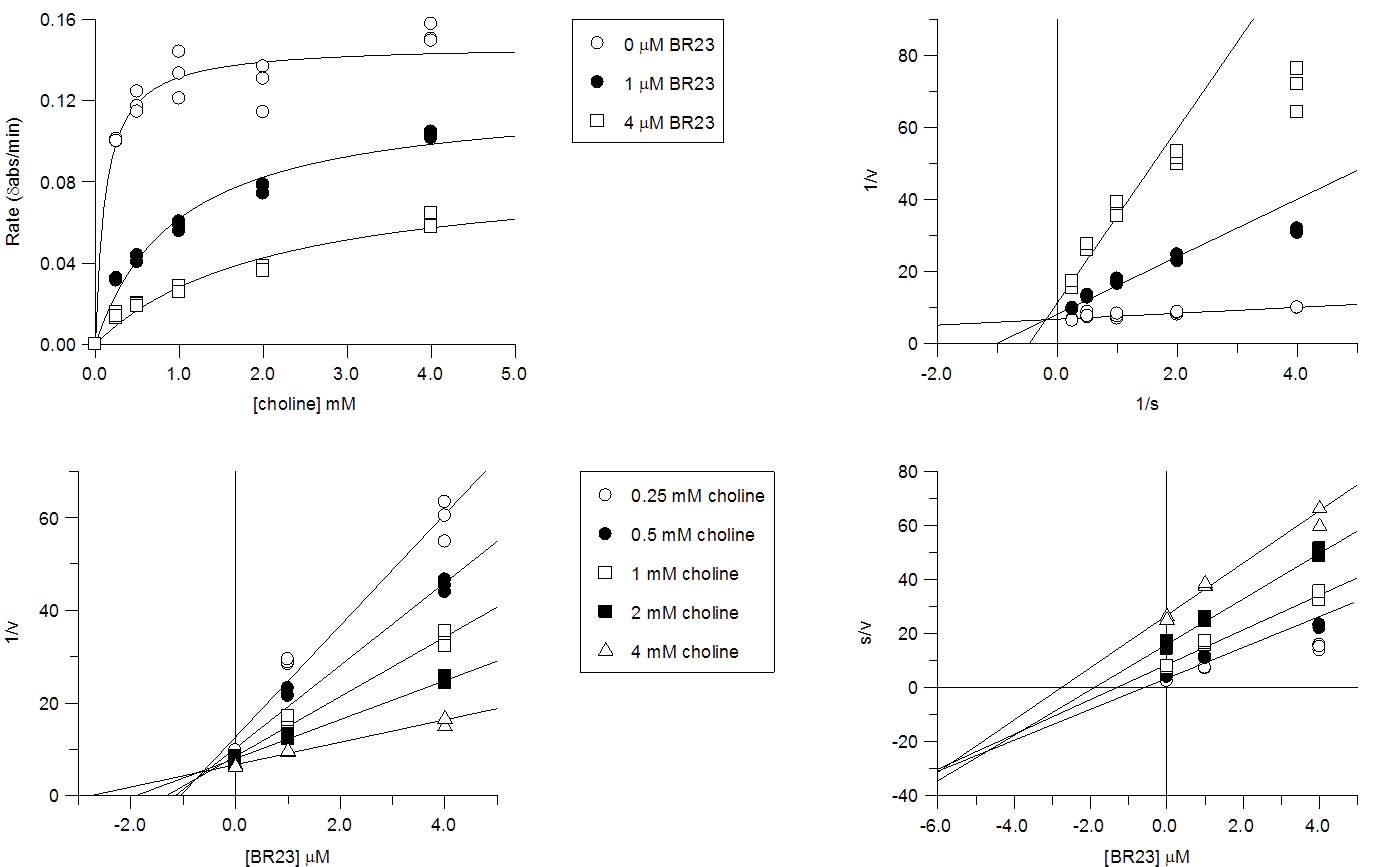


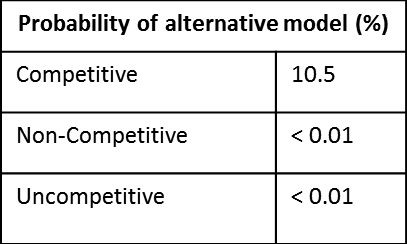


**S6 Fig. Inhibition kinetics of *Hs*CKα1by BR23, ATP as variable substrate**

**
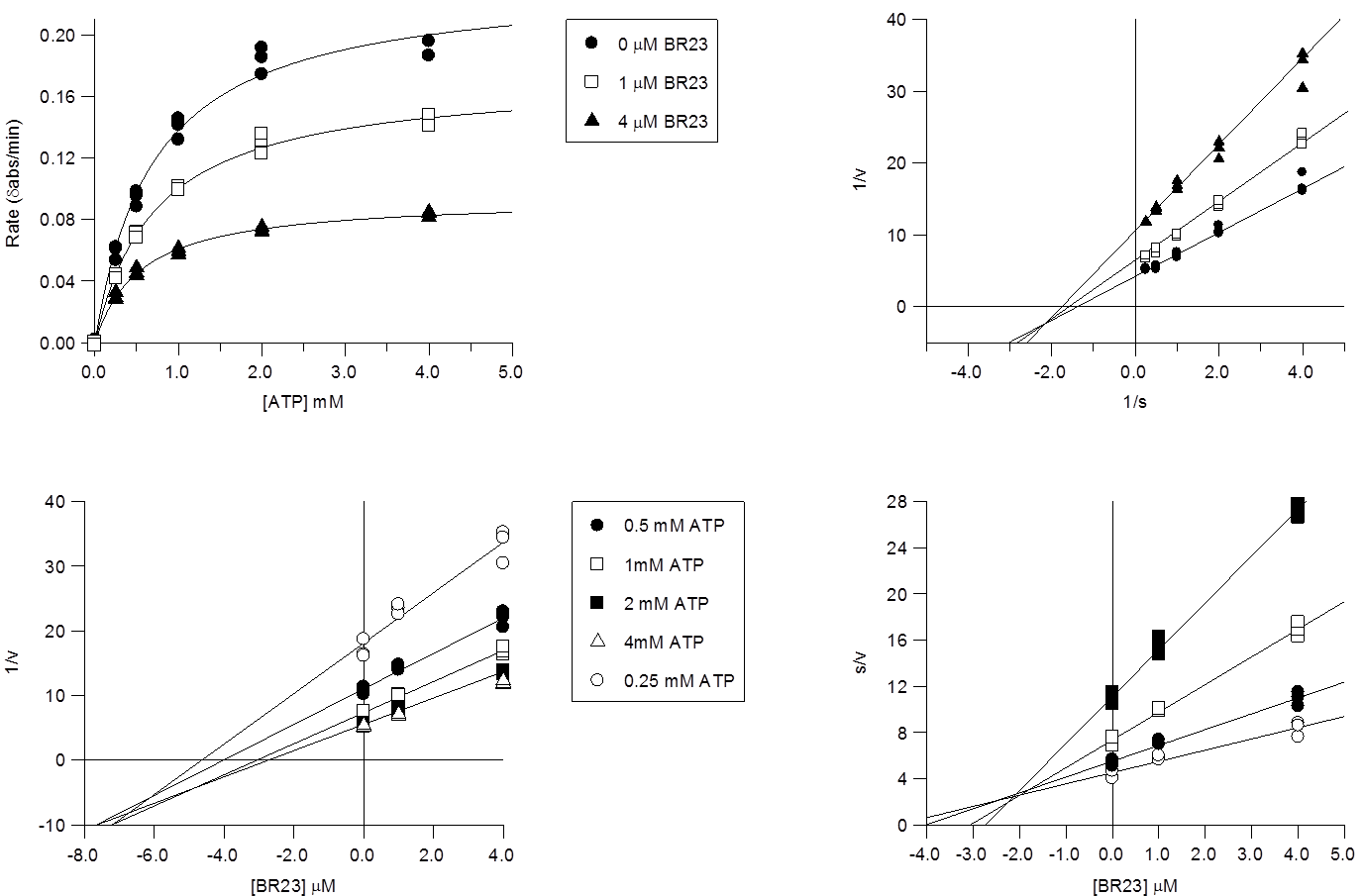
**


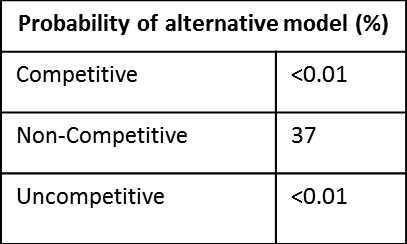


**S7 Fig. Inhibition kinetics of *Hs*CKα1 by BR25, Cho as variable substrate**

**
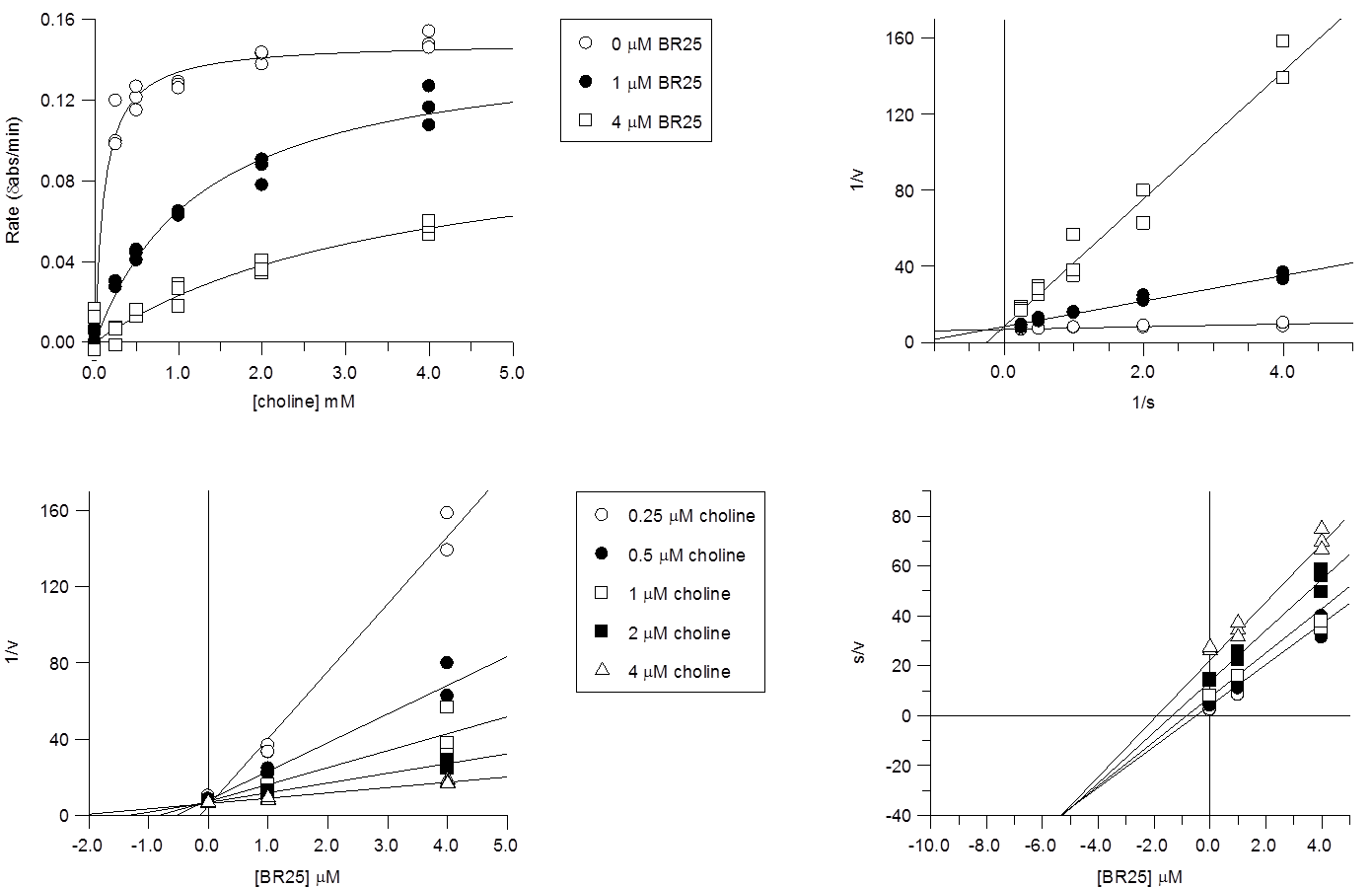
**

**
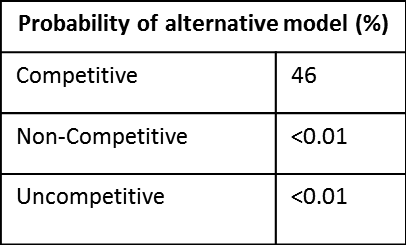
**

**S8 Fig. Inhibition kinetics of *Hs*CKα1 by BR25, ATP as variable substrate**

**
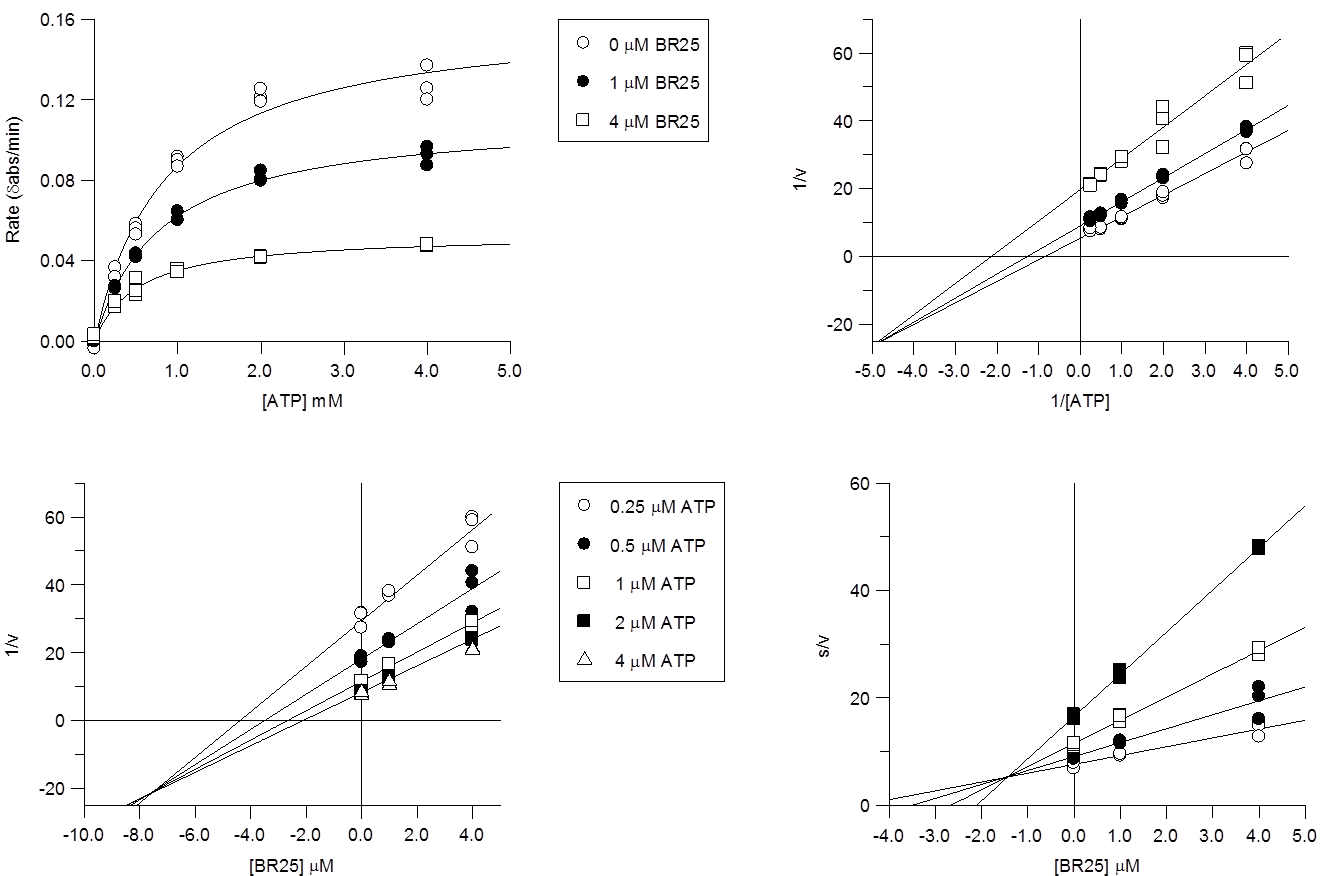
**


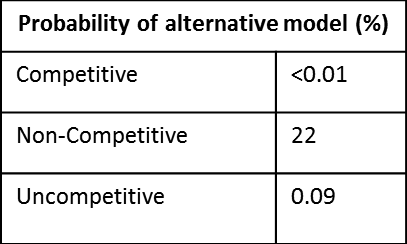


**S9 Fig. Inhibition kinetics of *Pf*CK by BR25, Cho as variable substrate**


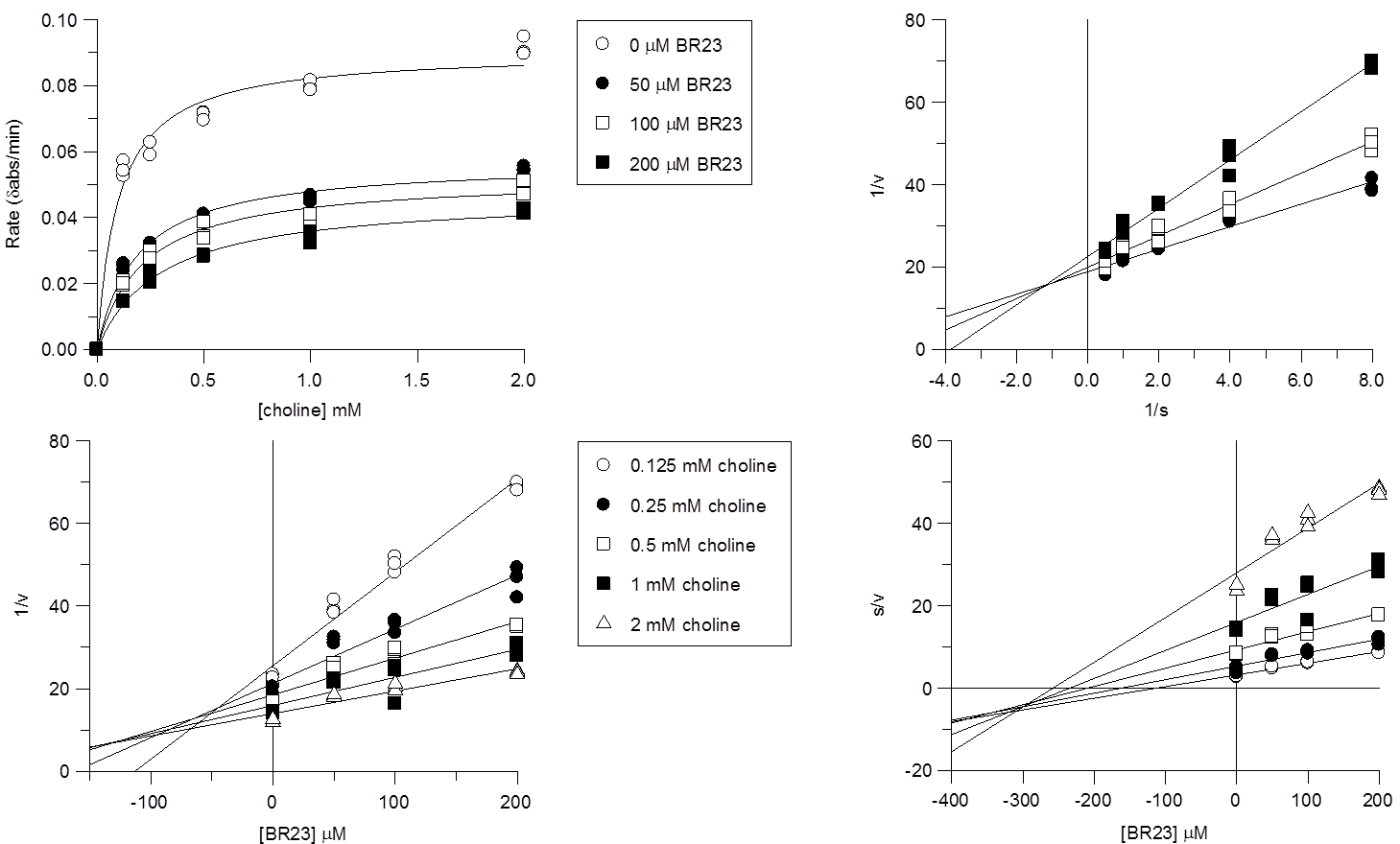


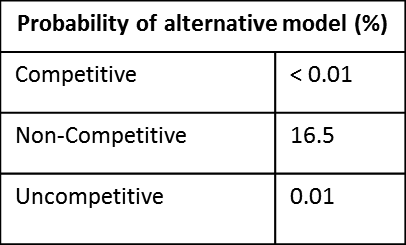


**S10 Fig. Quenching of intrinsic *Hs*CKα1 and *Pf*CK tryptophan fluorescence measured at increasing concentrations of compounds BR23 and BR25.** All data points represent the means ± S.D. for three measurements. The K_d_ for compounds BR23 and BR25 was determined by fitting fluorescence intensity data against their concentrations (values showed in table below).

**
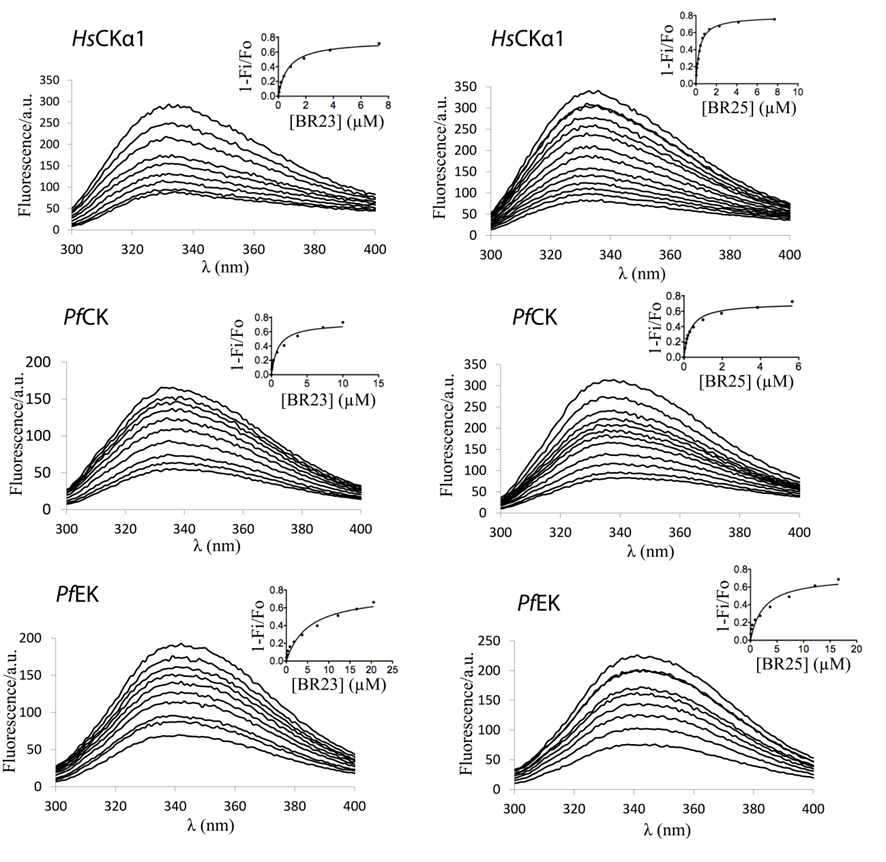
**

**S11 Fig. Stereo view of the HsCKα1 choline-binding site bound to BR25.** Electron density map is F_O_ − F_C_ contoured at 2.2 σ for BR25.


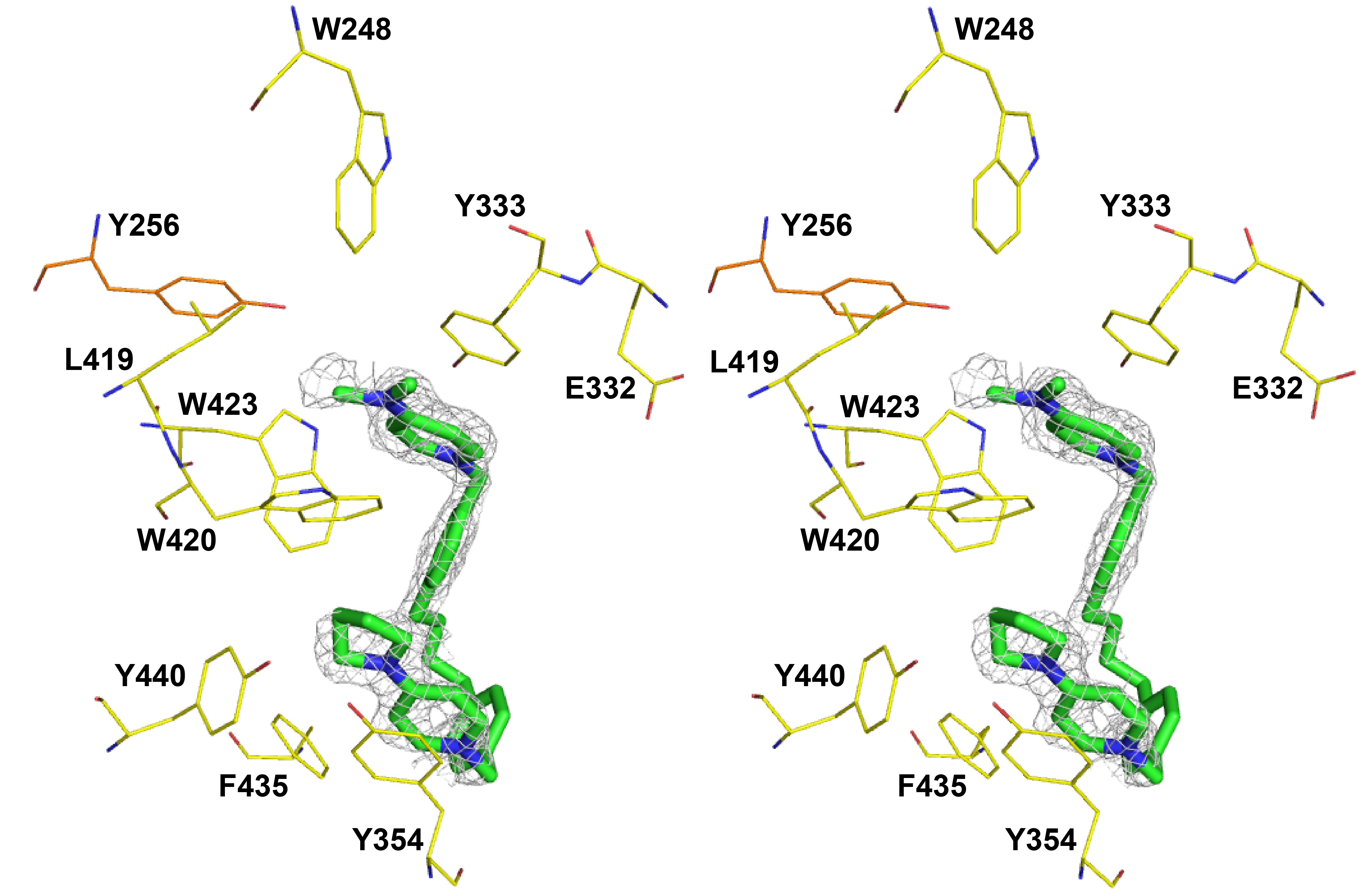


**SUPPLEMENTAL TABLES**

**S1 Table.** **IC_50_ values for inhibition of recombinant *Hs*CKα1, *Pf*CK and *Pf*EK by BR23 and BR25 in the presence of either Cho or Etn.**

|  | ***Hs*CK** | | ***Pf*EK** | | ***Pf*CK** | |
| --- | --- | --- | --- | --- | --- | --- |
|  | **Cho** | **Etn** | **Cho** | **Etn** | **Cho** | **Etn** |
| **BR23** | **0.58 µM** | **0.23 µM** | **N.A.** | **> 500 µM** | **276 µM** | **12 µM** |
| **BR25** | **0.56 µM** | **0.18 µM** | **N.A.** | **> 500 µM** | **103 µM** | **6 µM** |

N.A., not applicable

**S2 Table. K_d_ values for compounds BR23 and BR25 determined by tryptophan fluorescence quenching.**

|  | ***Hs*CKα1 (nM)** | ***Pf*CK**  **(nM)** |
| --- | --- | --- |
| **BR23** | 521 ± 279 | 1400 ± 423 |
| **BR25** | 352 ± 19 | 415 ± 81 |

**S3 Table. Data collection and refinement statistics:** Values in parentheses refer to the highest resolution shell. Ramachandran plot statistics were determined with PROCHECK.

|  | ***Hs*CKα1-**  **BR25** |
| --- | --- |
| Space group | P4_3_2_1_2 |
| Wavelength (Å) | 1.05 |
| Resolution (Å) | 20-1.60  (1.69-1.60) |
| Cell dimensions (Å) | *a =61.36*  *b* = 61.36  *c* = 219.71 |
| Unique reflections | 56719 |
| Completeness | 99.9 (100) |
| *R*_sym_ | 0.045 (0.673) |
| *I*/σ(*I*) | 44.2 (6.6) |
| Redundancy | 25.4 (26.2) |
| *R*_work_ / *R*_free_ | 0.180/0.205 |
| RMSD from ideal geometry, bonds (Å) | 0.007 |
| RMSD from ideal geometry, angles (º) | 1.065 |
| <*B*> protein (Å^2^) | 29.08 |
| <*B*> ligand (Å^2^) | 56.26 |
| <*B*> solvent (278 water molecules) (Å^2^) | 40.50 |
| <*B*> ethylene glycol (Å^2^) | 49.26 |
| Ramachandran plot:  Most favoured (%)  Additionally allowed (%)  Outliers (%) | 97.63  2.37  0.00 |
| PDB ID | 5FUT |
